# Efficacy and outcomes of BCG re-vaccination in COVID-19: a systematic review, meta-analysis, and meta-regression of randomized controlled trials

**DOI:** 10.1097/MS9.0000000000002370

**Published:** 2024-07-17

**Authors:** Hritvik Jain, Ramez M. Odat, Ayham Mohammad Hussein, Debankur Dey, Mushood Ahmed, Jyoti Jain, Aman Goyal, Tanya Ratnani, Muhammad Idrees, Priyadarshi Prajjwal, Siddhant Passey, Rukesh Yadav

**Affiliations:** a Department of Internal Medicine, All India Institute of Medical Sciences (AIIMS), Jodhpur; b Medical College Kolkata, Kolkata; c Department of Internal Medicine, Seth GS Medical College and KEM Hospital, Mumbai; d Department of Internal Medicine, Chhattisgarh Institute of Medical Sciences, Bilaspur; e Bharati Vidyapeeth Deemed University, Pune, India; f Faculty of Medicine, Jordan University of Science and Technology, Irbid; g Faculty of Medicine, Al-Balqa’ Applied University, Salt, Jordan; h Department of Internal Medicine, Rawalpindi Medical University, Rawalpindi; i Lahore General Hospital, Lahore, Pakistan; j Department of Internal Medicine, University of Connecticut Health Center, CT, USA; k Department of Internal Medicine, Maharajgunj Medical Campus, Institute of Medicine, Tribhuvan University, Nepal

**Keywords:** BCG vaccine, coronavirus, COVID-19, SARS-CoV-2, vaccine

## Abstract

**Introduction:**

The Bacillus Calmette-Guerin (BCG) vaccine has a beneficial “off-target” effect that offers heterologous protection against respiratory tract infections by inducing trained immunity. The need for producing antigen-specific COVID-19 vaccines leads to delays in vaccine administration. Current randomized controlled trials (RCTs) report conflicting data on BCG’s efficacy in COVID-19 infection.

**Methods:**

A comprehensive literature search was conducted using major bibliographic databases to identify RCTs evaluating the outcomes of BCG re-vaccination in COVID-19. For dichotomous outcomes, odds ratios (ORs) with 95% CIs were pooled using the DerSimonian-Laird random-effects model. Statistical significance was set at *P* less than 0.05.

**Results:**

Thirteen RCTs with 13 939 participants (7004 in the BCG re-vaccination group and 6935 in the placebo group) were included. BCG re-vaccination did not lead to a statistically significant difference in the incidence of COVID-19 infection [OR: 1.04; 95% CI: 0.91, 1.19; *P*=0.56], COVID-19-related hospitalizations [OR: 0.81; 95% CI: 0.38, 1.72; *P*=0.58), ICU admissions [OR: 0.43; 95% CI: 0.13, 1.46; *P*=0.18], or mortality [OR: 0.67; 95% CI 0.15, 3.04; *P*=0.60]. For safety outcomes, BCG re-vaccination led to a significant increase in the local injection site complications [OR: 99.79; 95% CI: 31.04, 320.80; *P*<0.00001], however, the risk of serious adverse events was similar [OR: 1.19; 95% CI: 0.84, 1.67; *P*=0.33].

**Conclusions:**

BCG re-vaccination does not decrease the incidence of COVID-19 infection, COVID-19-related hospitalizations, ICU admissions, COVID-19-related mortality, and serious adverse events; however, it leads to a rise in local injection site complications. Caution should be exercised when overstating BCG’s efficacy in COVID-19 prevention.

## Graphical abstract

**Figure:**
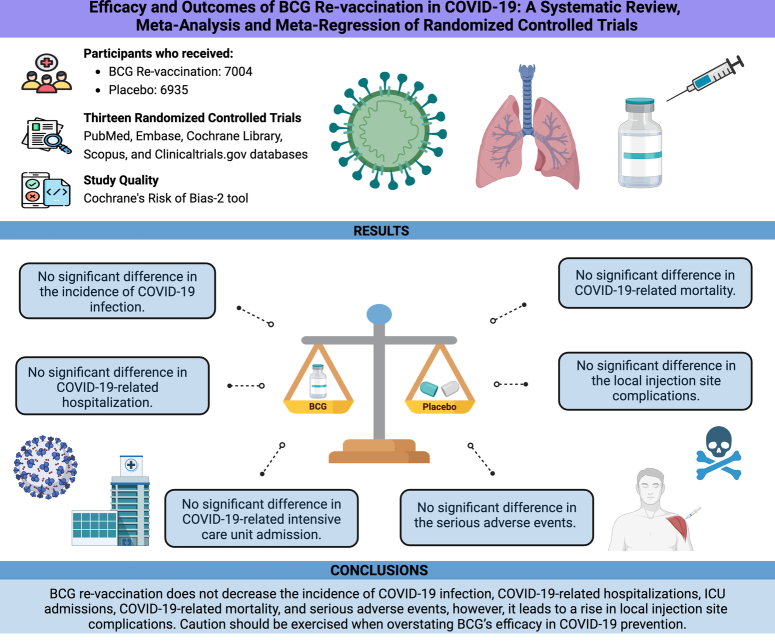

## Introduction

Highlights

- The Bacillus Calmette-Guerin (BCG) vaccine has a beneficial “off-target” effect of protecting against respiratory tract infections.
- BCG re-vaccination did not lead to a significant reduction in the incidence of COVID-19, COVID-19-related-hospitalizations, ICU admissions, and mortality.
- BCG re-vaccination did not increase the risk of serious adverse events but led to an increase in local injection site complications.
- Further large-scale randomized trials are warranted to evaluate the robustness of our results.

The global outbreak of the SARS-CoV-2 in 2019 led to a pandemic of the COVID-19, associated with detrimental morbidity and mortality^1^. The Bacillus Calmette-Guerin (BCG) vaccine was introduced in the early 1900s and is the sole vaccination against tuberculosis^2^. In addition to providing immunity against tuberculosis, the BCG vaccine has been demonstrated to have certain “off-target” immunomodulatory effects that provide trained immunity against various other infections^3–6^. BCG vaccine is associated with a reduced risk of all-cause mortality in infants, a reduced risk of respiratory tract infections and yellow fever in healthy adults^4,5,7–9^. These effects of the BCG vaccine are attributable to the ability to modify the immune thresholds through various mechanisms including heterologous T-cell immunity and immune cell reprogramming^5,10–12^. These properties of the BCG vaccine might be beneficial in COVID-19 infection by increasing antiviral immunity^13^. Since SARS-CoV-2 displays antigenic shifts which leads to the development of escape mutants, previous vaccines for COVID-19 are rendered useless against the newer antigenic strains. Hence, there is a delay between novel strain detection and mass administration of the new vaccine, which is associated with increased mortality^10,14^. To bridge this gap, it is hypothesized that BCG vaccines might render non-specific immunity against COVID-19. However, the current literature displays inconsistent results, with some studies demonstrating a reduction in COVID-19-related mortality and morbidity in countries with widespread BCG national immunization programs^15^. Contrastingly, Arlehamn *et al.* ^16^ concluded insignificant results in COVID-19-related mortality outcomes with BCG administration. The largest and most recently published multinational trial by Pittet *et al.* ^17^ concluded an insignificant reduction of COVID-19 infection risk among healthcare workers re-vaccinated with the BCG vaccine. To comprehensively evaluate and assess the efficacy of BCG re-vaccination in COVID-19 disease, we conducted a meta-analysis of randomized controlled trials, including the most recently published BRACE trial.

## Materials and methods

This current systematic review and meta-analysis followed the guidelines put forth by the Preferred Reporting Items for Systematic Review and Meta-Analysis Statement (PRISMA 2020) (Supplementary Digital Content 1, http://links.lww.com/MS9/A556)^18^. The study protocol was registered at the International PROSPERO Registry before the initiation of this review (CRD42024521390). This work has been reported following the AMSTAR guidelines (Supplementary Digital Content 2, http://links.lww.com/MS9/A557)^19^.

### Search strategy

An electronic search spanning the major literature databases such as Medline (via PubMed), Embase, the Cochrane Library, Scopus, and the International Registry of Clinical Trials (www.clinicaltrials.gov) was done, from their inception up to April 2024. A search strategy was constructed using a combination of medical subject heading (MeSH) and keywords including: “BCG vaccine”, “bacillus calmette guerin”, “BCG”, “COVID-19”, “SARS-CoV-2”, and “coronavirus”. We used a combination of Boolean operators like “AND” and “OR” to create the search strategy. The search strategy was modified according to specific databases. The detailed search strategy is depicted in Supplementary Table S1, Supplemental Digital Content 3, http://links.lww.com/MS9/A558. In addition to this, the reference list of the included articles, reviews, and previous meta-analyses were scrutinized to identify potential records. No restrictions were imposed on the publication year and the language of publication.

### Study selection and eligibility criteria

We examined studies that adhered to the following inclusion criteria: (i) randomized controlled trials (RCTs), (ii) one group was re-vaccinated with BCG, (iii) one group was administered placebo/control, (iv) investigated at least one of the desired outcomes: incidence of COVID-19 infection, COVID 19 related hospitalization, COVID-19-related ICU admission, COVID-19-related mortality, safety of BCG vaccine outcomes like local injection response, and serious adverse events. Trials were considered irrespective of geographical location and racial background. We excluded observational studies, review articles, editorials, case reports, viewpoints, and correspondences.

### Data extraction and quality assessment

The shortlisted articles retrieved from the literature search underwent removal of duplicates using the EndNote Reference Library X7 software (Clarivate Analytics). The remaining records were scrutinized by two investigators (H.J. and A.M.H.), and any discrepancy in judgment was resolved by involving a third investigator (R.M.O.). Data extracted from each RCT included: author name, year, study design, country, number of participants, age, male sex, and the BCG vaccine strain.

For RCTs, each trial was evaluated for risk of bias using Cochrane’s Risk of Bias-2 (RoB-2) tool^20^. RoB-2 tool scrutinizes trials on several domains, including the randomization process, deviations from intended intervention, missing outcome data, measurement of outcome, and selection of reported results. Traffic plots and summary plots were created for a visual depiction of the analysis. Two investigators conducted this quality assessment and risk of bias examination (D.D. and H.J.), whereas, in cases of discrepancy, a third author was consulted (R.M.O.).

### Data synthesis

For all statistical analyses in this meta-analysis, Review Manager Version 5.4 (Nordic Cochrane Center, The Cochrane Collaboration, Copenhagen, Denmark), OpenMeta Analyst, and R-software were used. The results from all the included studies were presented as odds ratio (OR) with a 95% CI. Assuming variability in the studies included, the DerSimonian and Laird random-effects model was utilized to pool the outcomes and create forest plots. To confirm the statistical significance of the results, a *p* value of less than 0.05 was considered.

A meta-regression analysis was also conducted for all the outcomes to assess the impact of the mean age in the BCG re-vaccination group and publication year on the pooled estimates. For a visual depiction of meta-regression analysis, meta-regression bubble plots were generated to visualize the results. To explore heterogeneity across the studies, the Higgins I2 metric was utilized and a value less than 50% was deemed acceptable^21,22^. For outcomes with an I^2^ value greater than 50%, a sensitivity analysis was conducted using the “leave-one-out method” to identify the study contributing most to the heterogeneity. For assessing publication bias, a visual inspection of funnel plots, Egger’s regression test, and Begg-Mazumdar’s rank correlation tests were conducted^23,24^. For Egger’s regression test and Begg-Mazumdar’s rank correlation test, a *p* value of greater than 0.05 was considered insignificant publication bias. We conducted a subgroup analysis by categorizing based on the type of BCG vaccine strain.

## Results

### Study selection

The electronic literature search yielded a total of 1785 potentially relevant articles. Following deduplication (*n*=829), 956 articles were subjected to title and abstract screening and 758 were excluded. Subsequently, a total of 198 articles were subjected to a more comprehensive full-text assessment, and 185 were excluded due to various reasons: wrong outcomes (*n*=115), wrong study design (*n*=46), and wrong publication type (*n*=24). Finally, 13 studies were included in this meta-analysis^17,25–36^. This process of study selection is depicted in the PRISMA flowchart (Supplementary Figure S1, Supplemental Digital Content 3, http://links.lww.com/MS9/A558).

### Study and patient characteristics

A total of 13 939 participants were included in the final analysis, 7004 in the BCG vaccine group and 6935 in the control group. The publication year of studies ranged from 2021 to 2023. Three studies were conducted in the Netherlands^27,29,32^, one in Brazil^25^, one in Germany^26^, one in Poland^28^, one in the United States of America^30^, one in Denmark^31^, one in Mexico^33^, one in India^34^, one in Greece^35^, one in South Africa^36^, and one multicentric study involving Australia, Netherlands, Spain, United Kingdom and Brazil^17^. The baseline characteristics of the studies are depicted in Table 1.

**Table 1:** Baseline characteristics of included studies.

			No. participants; *n*		Age; [mean±SD] or [median (IQR)]		Males; %		
Author name (year)	Study design	Country	BCG	Placebo	BCG	Placebo	BCG	Placebo	BCG strain
Anjos *et al*.^25^	RCT	Brazil	64	67	41.8±11.0	44.2±11.3	31.3	16.4	BCG Moscow
Blossey *et al*.^26^	RCT	Germany	1013	1012	67.2±5.5	67.5±5.5	53.1	52.8	BCG Prague (VPM1002)
Claus *et al*.^27^	RCT	Netherlands	665	644	41.79±12.66	43.21±12.73	24.6	26.6	BCG Danish strain 1331
Czajka *et al*.^28^	RCT	Poland	168	174	46.3±12.1	44.7±11.8	24.4	13.8	BCG-10 vaccine
Doesschate *et al*.^29^	RCT	Netherlands	753	758	41.3±12.6	42.8±12.7	24	27.4	BCG Danish strain 1331
Faustman *et al*.^30^	RCT	United States of America	96	48	39.3±1.3	38.4±1.9	38.2	20.1	BCG Tokyo-172 strain
Madsen *et al*.^31^	RCT	Denmark	610	611	48 (37–56)	47 (36–57)	17	17	BCG Danish strain 1331
Moorlag *et al*.^32^	RCT	Netherlands	1008	1006	67 (64–72)	67 (64–72)	51.2	53.9	BCG Danish strain 1331
Pittet *et al*.^17^	RCT	Australia, Netherlands, Spain, United Kingdom, and Brazil	1703	1683	42.8±12.0	42.8±12.0	26.9	23.9	BCG-Denmark vaccine
Ramos-Martinez *et al*.^33^	RCT	Mexico	30	30	38 (28–54)	42 (33–49)	30	20	BCG Pasteur Mériux Connaught
Sinha *et al*.^34^	RCT	India	246	249	43±10	44±10	50	54	BCG Moscow strain
Tsilika *et al*.^35^	RCT	Greece	148	153	68.6±10.4	68.7±10.6	66.2	69.7	BCG Moscow strain 361-I
Upton *et al*.^36^	RCT	South Africa	500	500	39 (30-49)	39 (30-50)	30.2	29.0	BCG Danish strain 1331

BCG, Bacillus Calmette-Guerin; IQR, interquartile range; RCT, randomized controlled trials.

### Outcomes

#### Incidence of COVID-19 infection

**Figure 1:**
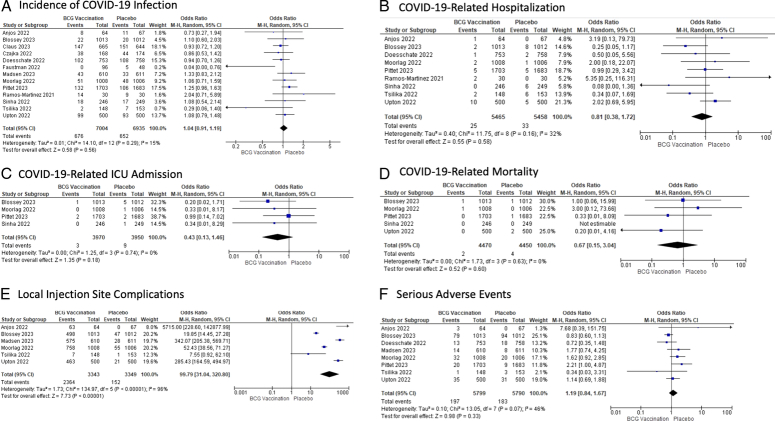
Forest plots for pooled analyses comparing BCG re-vaccination to placebo. The odds ratios (ORs) with their 95% CIs are depicted on a logarithmic scale. The diamond symbolizes the combined or overall effect. (A) Incidence of COVID-19 infection; (B) COVID-19-related hospitalization; (C) COVID-19-related ICU admission; (D) COVID-19-related mortality; (E) Local injection site complications; (F) Serious adverse events. BCG, Bacillus Calmette-Guerin.

Data on the incidence of COVID-19 infection was reported in all studies^17,25–36^. BCG re-vaccination did not lead to a statistically significant difference in the incidence of COVID-19 infection [OR: 1.04; 95% CI: 0.91, 1.19; *P*=0.56; I^2^=15%] than the placebo group (Fig. 1A). Meta-regression analysis was insignificant for the mean age of the BCG re-vaccination group (coefficient=−0.000; lower bound=−0.014; upper bound=0.013; *P*=0.950) (Supplementary Figure S2, Supplemental Digital Content 3, http://links.lww.com/MS9/A558) and publication year as a covariate (coefficient=0.073; lower bound=−0.15; upper bound=0.297; *P*=0.521) (Supplementary Figure S3, Supplemental Digital Content 3, http://links.lww.com/MS9/A558). Publication bias was insignificant on Egger’s test (*P*=0.615) and Begg-Mazumdar’s test (*P*=0.968) (Supplementary Figure S4, Supplemental Digital Content 3, http://links.lww.com/MS9/A558).

#### COVID-19-related hospitalization

Data on COVID-19-related hospitalization was reported in 9 studies^17,25,26,29,32–36^. BCG re-vaccination did not lead to a statistically significant difference in COVID-19-related hospitalization [OR: 0.81; 95% CI: 0.38, 1.72; *P*=0.58; I^2^=32%] than the placebo group (Fig. 1B). Meta-regression analysis was insignificant for the mean age of the BCG re-vaccination group (coefficient=−0.045; lower bound=−0.091; upper bound=0.001; *P*=0.053) (Supplementary Figure S5, Supplemental Digital Content 3, http://links.lww.com/MS9/A558) and publication year as a covariate (coefficient=−0.732; lower bound=−1.892; upper bound=0.428; *P*=0.216) (Supplementary Figure S6, Supplemental Digital Content 3, http://links.lww.com/MS9/A558). Publication bias was insignificant on Egger’s test (*P*= 0.513) and Begg-Mazumdar’s test (*P*= 0.94) (Supplementary Figure S7, Supplemental Digital Content 3, http://links.lww.com/MS9/A558).

#### COVID-19-related ICU admission

Data on COVID-19-related ICU admission was reported in 4 studies^17,26,32,34^. BCG re-vaccination did not lead to a statistically significant difference in COVID-19-related ICU admission [OR: 0.43; 95% CI: 0.13, 1.46; *P*=0.18; I^2^=0%] than the placebo group (Fig. 1C). Meta-regression analysis was insignificant for the mean age of the BCG re-vaccination group (coefficient=−0.047; lower bound=−0.147; upper bound=0.053; *P*=0.357) (Supplementary Figure S8, Supplemental Digital Content 3, http://links.lww.com/MS9/A558) and publication year as a covariate (coefficient=0.356; lower bound=−2.333; upper bound=3.045; *P*=0.795) (Supplementary Figure S9, Supplemental Digital Content 3, http://links.lww.com/MS9/A558). The funnel plot had minor concerns about publication bias (Supplementary Figure S10, Supplemental Digital Content 3, http://links.lww.com/MS9/A558).

#### COVID-19-related mortality

Data on COVID-19-related mortality was reported in 5 studies^17,26,32,34,36^. BCG re-vaccination did not lead to a statistically significant difference in COVID-19-related mortality [OR: 0.67; 95% CI: 0.15, 3.04; *P*=0.60; I^2^=0%] than the placebo group (Fig. 1D). Meta-regression analysis was insignificant for the mean age of the BCG re-vaccination group (coefficient=−0.030; lower bound=−0.121; upper bound=0.061; *P*=0.518) (Supplementary Figure S11, Supplemental Digital Content 3, http://links.lww.com/MS9/A558) and publication year as a covariate (coefficient=−0.23; lower bound=−3.074; upper bound=2.614; *P*=0.874) (Supplementary Figure S12, Supplemental Digital Content 3, http://links.lww.com/MS9/A558). Publication bias was insignificant on Egger’s test (*P*=0.1242) and Begg-Mazumdar’s test (*P*=0.997) (Supplementary Figure S13, Supplemental Digital Content 3, http://links.lww.com/MS9/A558).

#### Local injection site complications

Data on the local injection site complications were reported in 6 studies^25,26,31,32,35,36^. BCG re-vaccination did lead to a statistically significant increase in the risk of local injection site complications [OR: 99.79; 95% CI: 31.04; 320.80; *P*<0.00001; I^2^=96%] than the placebo group (Fig. 1E). To evaluate the high heterogeneity (I^2^=96%), a sensitivity analysis using the “leave-one-out” method was carried out. Removal of Blossey 2023 decreased the I^2^ to 94%, however, all the studies seemed to influence the overall outcome (Supplementary Figure S14, Supplemental Digital Content 3, http://links.lww.com/MS9/A558). Meta-regression analysis was significant for the mean age of the BCG re-vaccination group (coefficient=−0.099; lower bound=−0.138; upper bound=−0.060; *P*<0.001) (Supplementary Figure S15, Supplemental Digital Content 3, http://links.lww.com/MS9/A558), but insignificant for publication year as a covariate (coefficient=−0.377; lower bound=−3.130; upper bound=2.375; *P*=0.788) (Supplementary Figure S16, Supplemental Digital Content 3, http://links.lww.com/MS9/A558). Publication bias was significant on Egger’s test (*P*<0.0001) but not on Begg-Mazumdar’s test (*P*=0.933) (Supplementary Figure S17, Supplemental Digital Content 3, http://links.lww.com/MS9/A558).

#### Serious adverse events

Data on serious adverse events were reported in 8 studies^17,25,26,29,31,32,35,36^. BCG re-vaccination did not lead to a statistically significant difference in the risk of serious adverse events [OR: 1.19; 95% CI: 0.84, 1.67; *P*=0.33; I^2^=46%] compared to the placebo group (Fig. 1F). Meta-regression analysis was insignificant for the mean age of the BCG re-vaccination group (coefficient=−0.056; lower bound=−0.198; upper bound=0.086; *P*=0.438) (Supplementary Figure S18, Supplemental Digital Content 3, http://links.lww.com/MS9/A558) and publication year as a covariate (coefficient=0.043; lower bound=−0.567; upper bound=0.653; *P*=0.889) (Supplementary Figure S19, Supplemental Digital Content 3, http://links.lww.com/MS9/A558). Publication bias was significant on Egger’s test (*P*=0.4571) but not on Begg-Mazumdar’s test (*P*=0.956) (Supplementary Figure S20, Supplemental Digital Content 3, http://links.lww.com/MS9/A558).

### Subgroup analysis

Subgroup analysis was conducted by dividing RCTs into BCG Danish strain 1331 versus other strains. No significant difference in the effect estimates was noted in the subgroup analysis for all six outcomes [Supplementary Figure S21-S26, Supplemental Digital Content 3, http://links.lww.com/MS9/A558].

### Quality assessment

Cochrane’s RoB-2 tool was utilized was used for risk of bias assessment [Supplementary Figure S27, S28, Supplemental Digital Content 3, http://links.lww.com/MS9/A558]. Out of the 13 trials, only Madsen 2023 and Tsilika 2022 were at high risk of bias, rest were either at low risk or had some concerns of bias.

## Discussion

In this meta-analysis, we examined the clinical outcomes of BCG re-vaccination using data from 13 RCTs involving 13 939 participants (7004 in the BCG vaccine group and 6935 in the control group). Our analysis did not observe any statistically significant reduction in COVID-19 infection, COVID-19-related hospitalization, COVID-19-related ICU admission, or COVID-19-related mortality. Additionally, BCG re-vaccination was associated with a significantly higher incidence of local injection site complications; however, it did not increase the risk of serious adverse events. On meta-regression analysis, the mean age of the BCG re-vaccination group as a covariate was significant in the pooled estimates for local injection site complications. Overall, BCG re-vaccination was found to be safe but did not confer significant protective benefits against SARS-CoV-2 infection. To the best of our knowledge, this meta-analysis is the largest to date, comprehensively evaluating BCG re-vaccination’s efficacy and safety by incorporating newer RCTs, conducting meta-regression analysis, as well as identifying publication bias.

Numerous studies have demonstrated the heterologous beneficial effects of the BCG vaccine against unrelated viral pathogens, such as herpes virus, influenza A (H1N1), and human papillomavirus^37,38^. This led to the hypothesis that the BCG vaccine might confer protection against COVID-19 due to its non-specific beneficial effects^39^. SARS-CoV-2, a single-stranded enveloped RNA virus, utilizes antigenic structural proteins known as spike glycoproteins to bind to the angiotensin-converting enzyme 2 (ACE2) type-II pneumocyte receptor, followed by its endocytosis and subsequent apoptotic damage to the host cells^40^. The release of RNA acts as a pathogen-associated molecular pattern (PAMP), triggering a surge of chemokines that leads to neutrophilic infiltration and consequent inflammatory damage^41^. While the specific mechanisms underlying the non-specific protective response of the BCG vaccine are still under investigation, Netea and colleagues described the effects of BCG vaccination on genome-wide histone modifications induced in trained monocytes, which were demonstrated to be associated with reduced counts of yellow fever virus viremia attributed to the increased IL-1β production^5^. The BCG vaccine effectively reprograms this innate immune response by inducing innate immune cells (NK cells, macrophages) and cells of the adaptive or ‘trained’ immunity (B- and T-cell responses) through epigenetic reprogramming of monocytes^42^. This trained immunity is associated with an elevation of pro-inflammatory cytokines, including TNF-alpha and interleukin-6, thereby enhancing antimicrobial responses, such as easier recognition of SARS-CoV-2 PAMP by pattern recognition receptors leading to increased cytokine production^43^. Metabolic reprogramming in favor of glycolysis further provides cofactors for epigenetic enzymatic activity^44^. A second mechanism proposed to contribute to the cross-protective effect of the BCG vaccine is heterologous immunity^45^. Specific amino acid sequences in the SARS-CoV-2 envelope protein have been found to have high homology to LytR C-terminal domain-containing proteins of the Mycobacterium species, which may activate heterologous immunity^46^. Similarly, Tomita *et al.* ^47^ identified similar 9-amino acid sequences between BCG and SARS-CoV-2. Furthermore, BCG vaccination can regulate anti-inflammatory cytokine and chemokine responses, potentially preventing hospitalization and higher severity of COVID-19 cases^48^. Indeed, epidemiological studies have indicated that countries with higher BCG vaccine coverage have lower rates of COVID-19 infection and mortality^7,49^. However, the non-specific immune benefits of the BCG vaccine may be clinically significant mainly in very young or old populations where response signals differ significantly^8,9^. Giamarellos-Bourboulis *et al.* ^6^ 2020 in the ACTIVATE clinical trial confirmed that BCG vaccination reduced the incidence of respiratory tract infections, suggesting a potential protective benefit against SARS-CoV-2 infection in the vulnerable population including healthcare workers.

Kaufmann and colleagues suggested that tissue tropism might play a role in explaining the lack of significant protective benefit of BCG re-vaccination. The investigators subcutaneously vaccinated Roborovski hamsters with the BCG vaccine, resulting in a significant lowering in morbidity and mortality against the H1N1 virus but not against SARS-CoV-2^50^. This failure was attributed to the unique pulmonary vasculature damage caused by the SARS-CoV-2 infection, facilitating viral spread to other organs, particularly the bone marrow, which is a crucial site for facilitating BCG-mediated trained immunity. Similarly, an observational study by Hilligan *et al.* ^51^ demonstrated that BCG administration intravenously, rather than the clinically used subcutaneous inoculation, protected human-ACE2 transgenic mice against the lethal challenge of SARS-CoV-2. Although intravenous BCG administration shows promising evidence in preventing COVID-19 infection in lab animals, it is not a clinically accepted practice currently^52^. Nonetheless, re-immunization with BCG may boost the efficacy of SARS-CoV-2 vaccines by boosting antibody and memory T-cell responses^53,54^. This suggests the possibility of using booster BCG as an adjunct to existing COVID-19 vaccines, thereby representing a therapeutic approach to increase their efficacy and promote the development of immunological memory. Given the absence of specific intervention measures during the early phases of the COVID-19 pandemic, innovative prophylactic approaches were crucial^39^.

With the ongoing evolution of the virus and the need for the development of strain-specific COVID-19 vaccines, a broad-spectrum approach is necessary to provide effective protection. Most studies conducted during the initial stage of the COVID-19 pandemic were observational, making interpretation challenging, it is theoretically possible to improve the efficacy of BCG vaccination by re-administering it as a homologous boost. Booster BCG vaccines have demonstrated protective effects and benefits against respiratory tract infections, particularly in the elderly population, and could potentially be cost-effective, considering the coverage of BCG in the immunization schedules of various countries^8,55^. However, the evidence on the value of BCG re-vaccination remains conflicting, and it is currently not recommended by the WHO, which is supported by the findings of this meta-analysis^9,56,57^.

### Future directions

Further research is necessary to elucidate where BCG re-vaccination protects against COVID-19 infection. It is conceivable that differences in testing and notification methodologies could obscure any distinctions in case and mortality rates of COVID-19 in countries with wide BCG vaccination. Hence, it is imperative to consider various factors such as age distribution, income level, rural-urban differences, socioeconomic level, and population density. Numerous ongoing trials are either underway or awaiting results^58–60^. Subsequent investigations should examine the optimal timing for BCG vaccination, the duration of its heterologous immunity, and its association with other injectable vaccines. The impact of vaccination age warrants careful consideration. Furthermore, randomized controlled trials are needed to clarify the hypothesis that trained immunity underlies the clinical benefits of BCG vaccination. Moreover, the therapeutic potential of BCG in conjunction with existing COVID-19 vaccines should be explored through rigorous clinical trials. Conclusive evidence regarding any protective effect of BCG vaccination must be established before informing clinical practice and vaccination strategies.

## Limitations

Although this current meta-analysis is the largest meta-analysis to date on this subject, it is important to interpret the results of this analysis with caution. Firstly, when performing this meta-analysis, we presumed that the participants’ baseline characteristics would be similar across all the studies. Secondly, most of the trials included in this meta-analysis were conducted on healthcare workers as subjects, which might restrict generalizing the results on persons in other diverse cohorts. Since healthcare workers are highly vulnerable to COVID-19 owing to higher exposure, the results might be biased. Thirdly, we could not account for the potential roles of the oral polio vaccine and measles-mumps-rubella vaccine, which are included in routine national immunization programs in various countries and may similarly induce “trained immunity” effects such as BCG^61^. Lastly, the results of the meta-regression are limited by the inclusion of the small number of available studies for some outcomes.

## Conclusions

BCG re-vaccination for COVID-19 infection is not associated with any significant benefits in clinical or safety outcomes including the incidence of COVID-19 infection, COVID-19-related hospitalization, COVID-19-related ICU admission, COVID-19-related mortality, local injection site complications, and serious adverse events. The findings of this meta-analysis do not support BCG vaccination in COVID-19 infection. However, more RCTs are warranted on this subject, taking into consideration the various strains of the BCG vaccine, and diversifying the patient population included.

## Ethical approval

Ethical approval was not required for this systematic review.

## Consent

Informed consent was not required for this systematic review.

## Source of funding

No funding received.

## Author contribution

H.J.: conceptualization, supervision, validation, visualization, writing—original draft, writing—review and editing; R.M.O.: formal analysis, project administration, investigation, writing—original draft, writing—review and editing; A.M.H.: resources, writing—original draft, writing—review and editing; D.D.: writing—original draft, writing—review and editing; M.A.: writing—original draft, writing—review and editing; J.J.: writing—original draft, writing—review and editing; A.G.: writing—original draft, writing—review and editing; T.R.: writing—original draft, writing—review and editing; M.I.: writing—original draft, writing—review and editing; P.P.: writing—original draft, writing—review and editing; S.P.: writing—original draft, writing—review and editing; R.Y.: project administration, writing—original draft, writing—review and editing.

## Conflicts of interest disclosure

The authors declare no conflicts of interest.

## Research registration unique identifying number (UIN)

PROSPERO Registration ID: CRD42024521390.

## Guarantor

Hritvik Jain.

## Data availability statement

Not applicable.

## Provenance and peer review

Not applicable.

## Supplementary Material
