# Supplementary material for: Efficacy and outcomes of BCG re-vaccination in COVID-19: a systematic review, meta-analysis, and meta-regression of randomized controlled trials
Source: Ann Med Surg (Lond). 2024 Jul 17;86(9):5439–46. doi: 10.1097/MS9.0000000000002370 (PMC11374198; doi:10.1097/MS9.0000000000002370)

**Supplementary Material**

**Efficacy and Outcomes of BCG Re-vaccination in COVID-19: A Systematic Review and Meta-Analysis**

**Supplementary Table S1**. Search strategies for online databases.

**Supplementary Figure S1**. The Preferred Reporting Items for Systematic Reviews and Meta-Analyses (PRISMA) 2020 flowchart.

**Supplementary Figure S2**. Meta-regression bubble plot showing the effect of the mean age of the BCG re-vaccination group on the incidence of COVID-19 infection.

**Supplementary Figure S3**. Meta-regression bubble plot showing the effect of publication year on the incidence of COVID-19 infection.

**Supplementary Figure S4**: Funnel plot for the incidence of COVID-19 infection.

**Supplementary Figure S5**: Meta-regression bubble plot showing the effect of the mean age of the BCG re-vaccination group on COVID-19-related hospitalization.

**Supplementary Figure S6**: Meta-regression bubble plot showing the effect of publication year on COVID-19-related hospitalization.

**Supplementary Figure S7**: Funnel plot for COVID-19-related hospitalization.

**Supplementary Figure S8**: Meta-regression bubble plot showing the effect of the mean age of the BCG re-vaccination group on COVID-19-related ICU admission.

**Supplementary Figure S9**: Meta-regression bubble plot showing the effect of publication year on COVID-19-related ICU admission.

**Supplementary Figure S10**: Funnel plot for COVID-19-related ICU admission.

**Supplementary Figure S11**: Meta-regression bubble plot showing the effect of the mean age of the BCG re-vaccination group on COVID-19-related mortality.

**Supplementary Figure S12**: Meta-regression bubble plot showing the effect of publication year on COVID-19-related mortality.

**Supplementary Figure S13**: Funnel plot for COVID-19-related mortality.

**Supplementary Figure S14**. Leave-one-out analysis for local injection site complications.

**Supplementary Figure S15**: Meta-regression bubble plot showing the effect of the mean age of the BCG re-vaccination group on local injection site complications.

**Supplementary Figure S16**. Meta-regression bubble plot showing the effect of publication year on local injection site complications.

**Supplementary Figure S17**. Funnel plot for local injection site complications.

**Supplementary Figure S18**: Meta-regression bubble plot showing the effect of the mean age of the BCG re-vaccination group on serious adverse events.

**Supplementary Figure S19**. Meta-regression bubble plot showing the effect of publication year on serious adverse events.

**Supplementary Figure S20**. Funnel plot for serious adverse events.

**Supplementary Figure S21**. Subgroup analysis for the incidence of COVID-19 infection.

**Supplementary Figure S22**. Subgroup analysis for COVID-19-related hospitalization.

**Supplementary Figure S23**. Subgroup analysis for COVID-19-related ICU admission.

**Supplementary Figure S24**. Subgroup analysis for COVID-19-related mortality.

**Supplementary Figure S25**. Subgroup analysis for local injection site complications.

**Supplementary Figure S26**. Subgroup analysis for serious adverse events.

**Supplementary Figure S27**. Cochrane’s Risk of Bias-2 (RoB-2) assessment for risk of bias in randomized controlled trials (RCTs).

**Supplementary Figure S28**. RoB-2 summary plot.

This supplemental material has been provided by the authors to give readers additional information about their work.

**Supplementary Table S1**. Search strategies for online databases

| Search Number | Database | Search Strategy | Number of Results |
| --- | --- | --- | --- |
| #1 | PubMed | ("BCG Vaccine"[Mesh] OR "BCG" OR "Bacillus calmette guerin") AND (("COVID-19"[MeSH]) OR ("SARS-CoV-2"[MeSH]) OR "coronavirus" OR "COVID19") | 594 |
| #2 | Embase | ("BCG Vaccine" OR "BCG" OR "Bacillus calmette guerin") AND (("COVID-19") OR ("SARS-CoV-2”) OR "coronavirus" OR "COVID19") | 464 |
| #3 | Cochrane | ((MeSH descriptor: [BCG Vaccine] explode all trees) OR (MeSH descriptor: [Mycobacterium bovis] explode all trees) OR (“BCG”)) AND ((MeSH descriptor: [COVID-19] explode all trees) OR “COVID-19” OR “COVID19” OR “SARS-CoV-2” OR “coronavirus”) | 122 |
| #4 | Scopus | (TITLE-ABS-KEY(“BCG vaccine”) OR TITLE-ABS-KEY(“bacillus calmette guerin”) OR TITLE-ABS-KEY(“BCG”)) AND (TITLE-ABS-KEY(COVID-19) OR TITLE-ABS-KEY(COVID19) OR TITLE-ABS-KEY(SARS-CoV-2) OR TITLE-ABS-KEY(coronavirus)) | 605 |
| TOTAL | | | 1785 |

**Supplementary Figure S1**. The Preferred Reporting Items for Systematic Reviews and Meta-Analyses (PRISMA) 2020 flowchart.


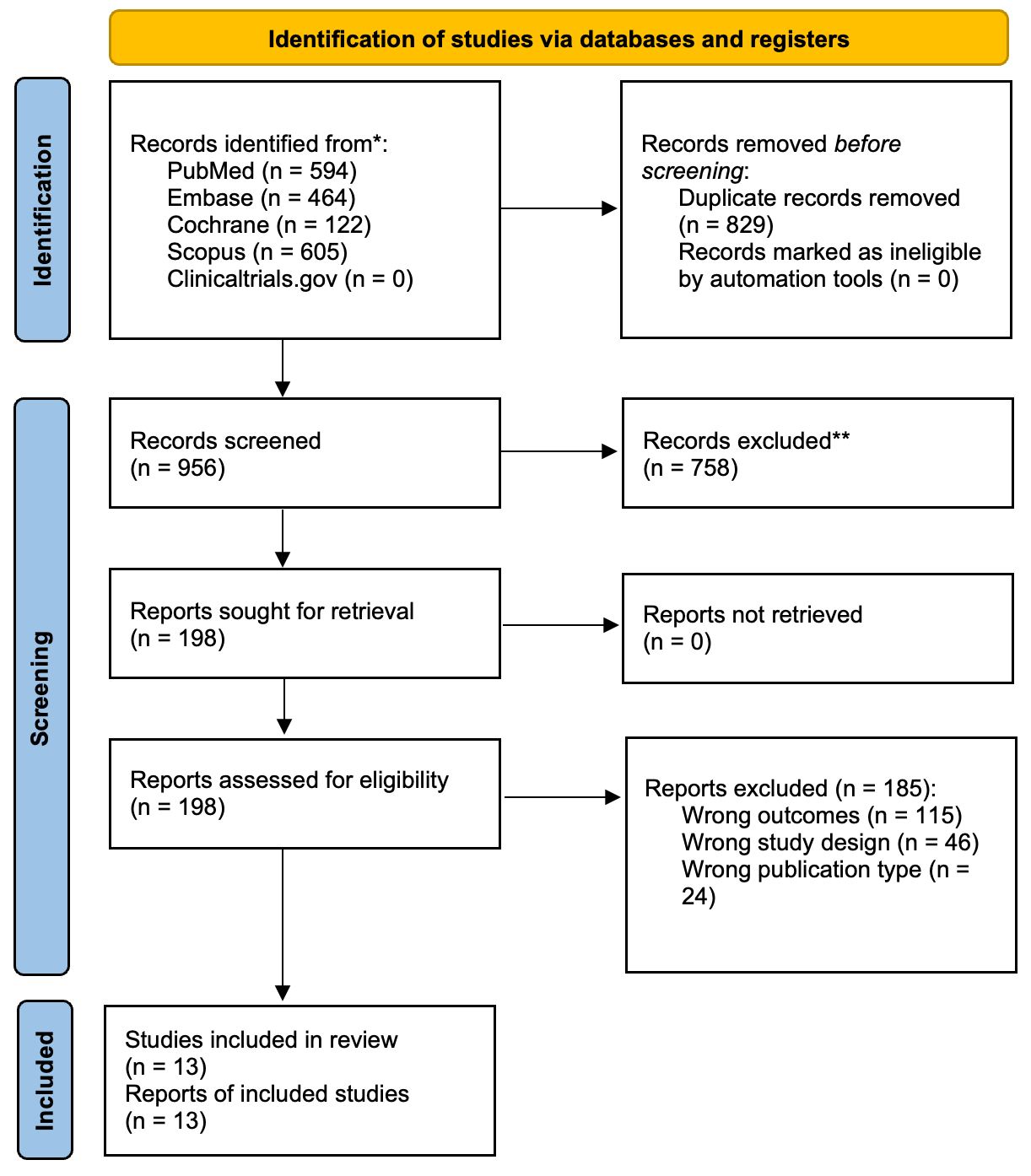


**Supplementary Figure S2**. Meta-regression bubble plot showing the effect of the mean age of the BCG re-vaccination group on the incidence of COVID-19 infection.


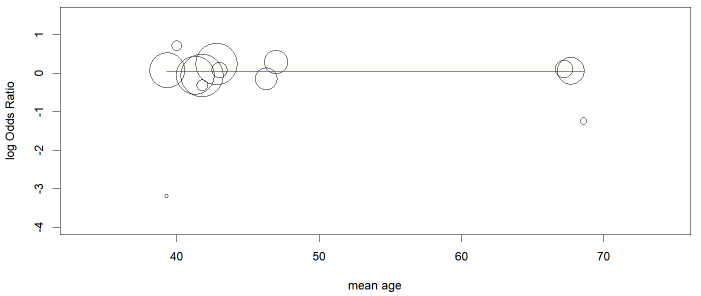


**Supplementary Figure S3**. Meta-regression bubble plot showing the effect of publication year on the incidence of COVID-19 infection.


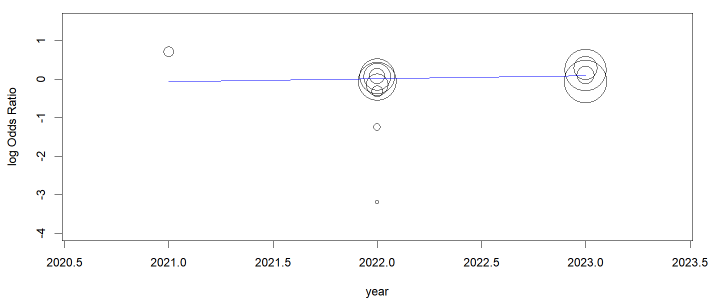


**Supplementary Figure S4**. Funnel plot for the incidence of COVID-19 infection.


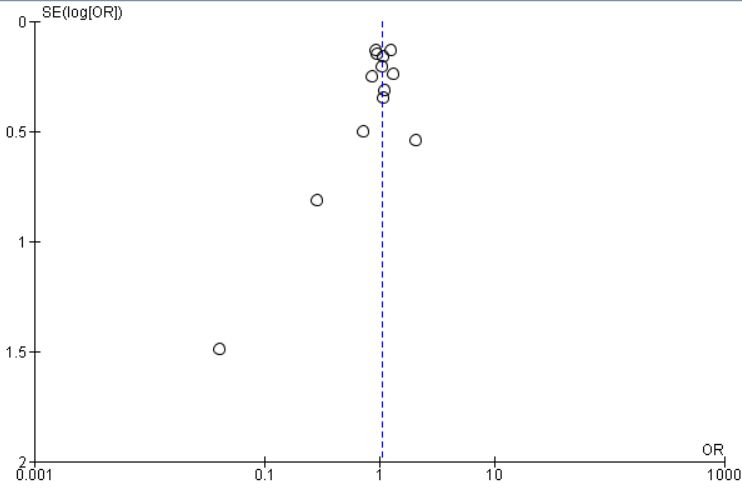


**Supplementary Figure S5**: Meta-regression bubble plot showing the effect of the mean age of the BCG re-vaccination group on COVID-19-related hospitalization.


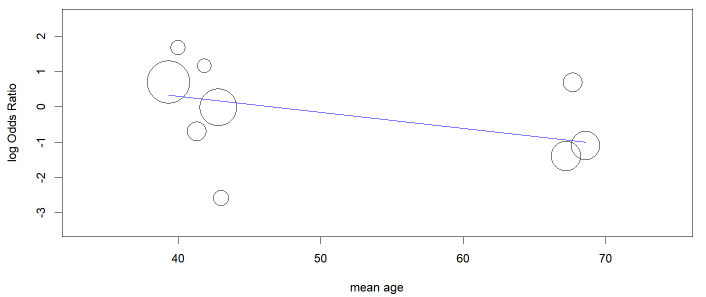


**Supplementary Figure S6**: Meta-regression bubble plot showing the effect of publication year on COVID-19-related hospitalization.


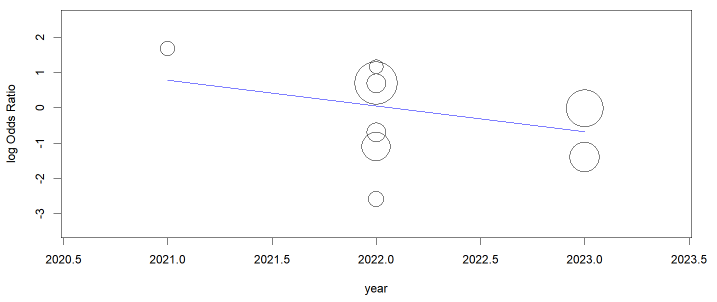


**Supplementary Figure S7**: Funnel plot for COVID-19-related hospitalization.


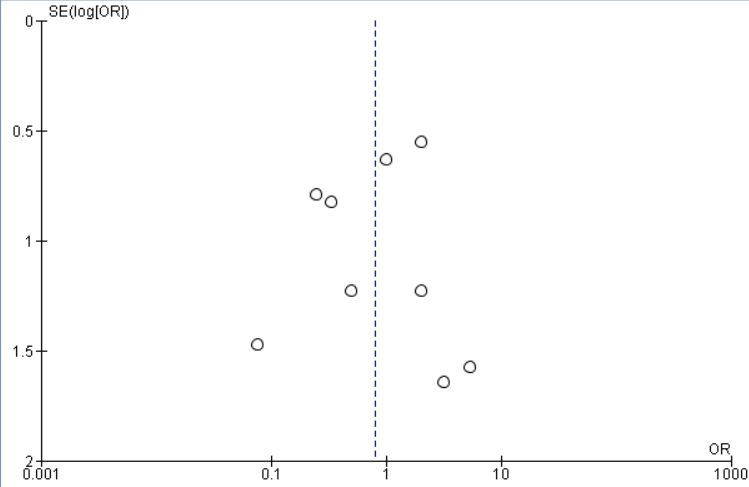


**Supplementary Figure S8**: Meta-regression bubble plot showing the effect of the mean age of the BCG re-vaccination group on COVID-19-related ICU admission.


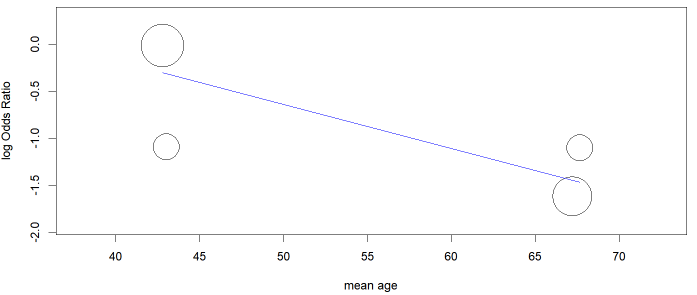


**Supplementary Figure S9**: Meta-regression bubble plot showing the effect of publication year on COVID-19-related ICU admission.


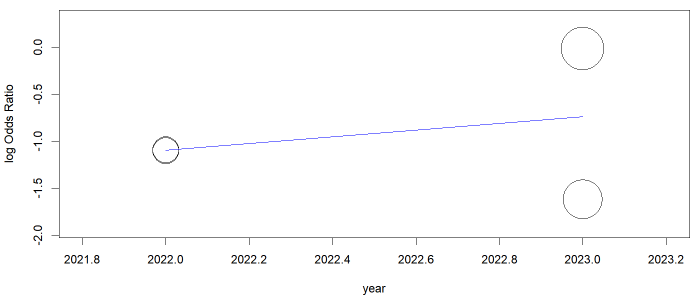


**Supplementary Figure S10**: Funnel plot for COVID-19-related ICU admission.


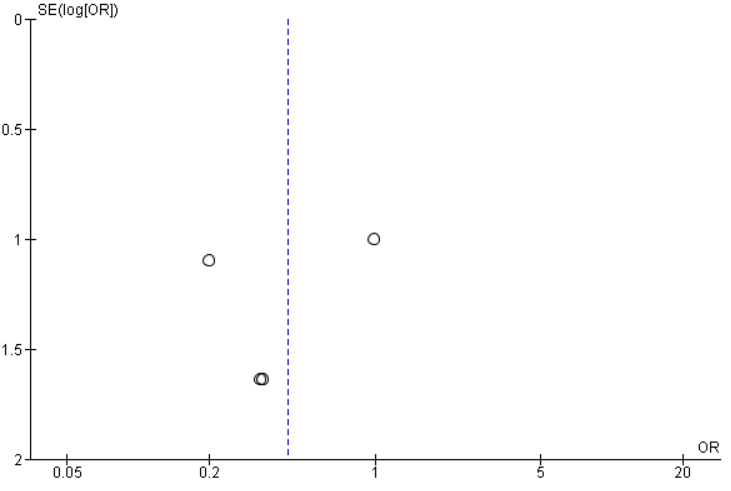


**Supplementary Figure S11**: Meta-regression bubble plot showing the effect of the mean age of the BCG re-vaccination group on COVID-19-related mortality.


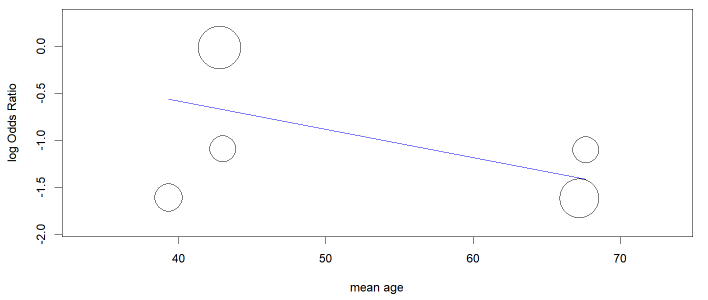


**Supplementary Figure S12**: Meta-regression bubble plot showing the effect of publication year on COVID-19-related mortality.


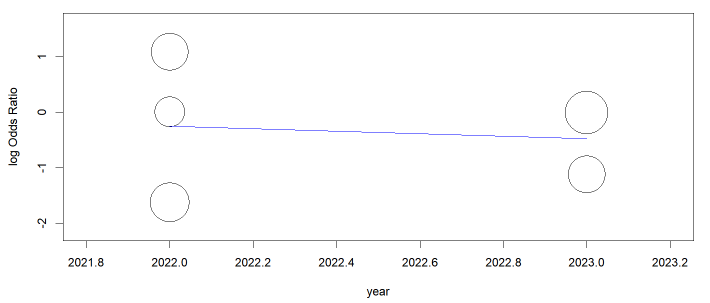


**Supplementary Figure S13**: Funnel plot for COVID-19-related mortality.

**
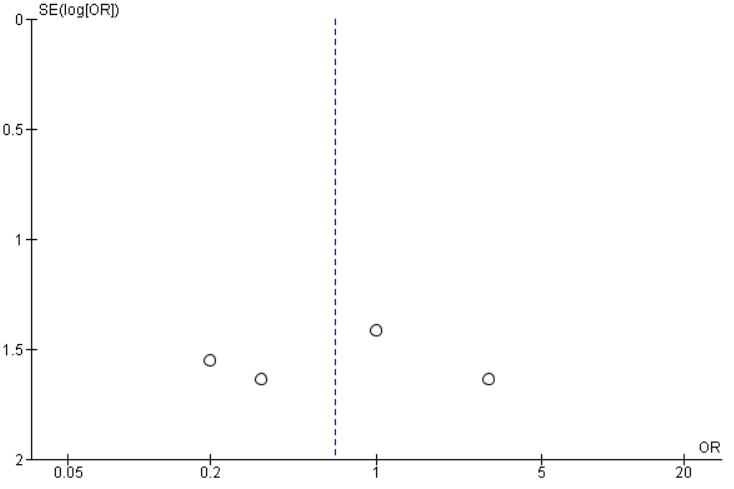
**

**Supplementary Figure S14**. Leave-one-out analysis for local injection site complications.


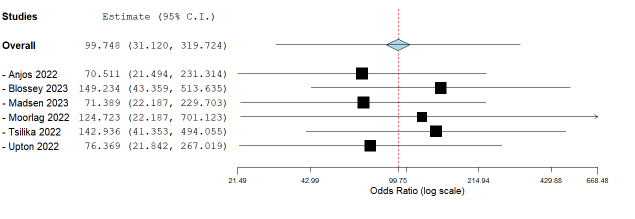


**Supplementary Figure S15**: Meta-regression bubble plot showing the effect of the mean age of the BCG re-vaccination group on local injection site complications.


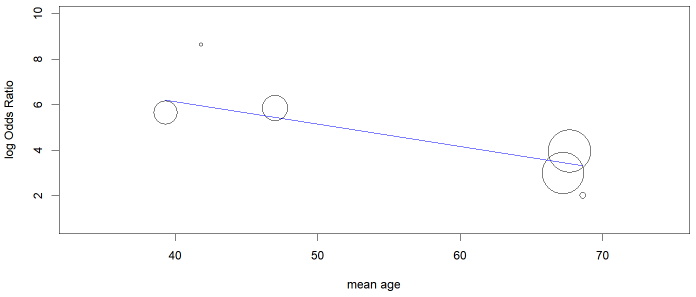


**Supplementary Figure S16**. Meta-regression bubble plot showing the effect of publication year on local injection site complications.


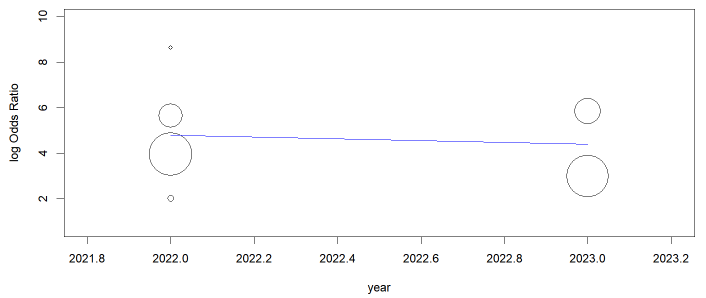


**Supplementary Figure S17**. Funnel plot for local injection site complications.


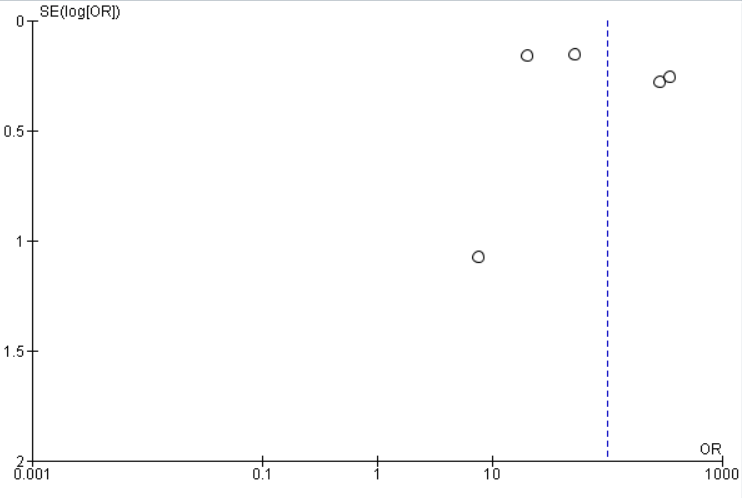


**Supplementary Figure S18**: Meta-regression bubble plot showing the effect of the mean age of the BCG re-vaccination group on serious adverse events.


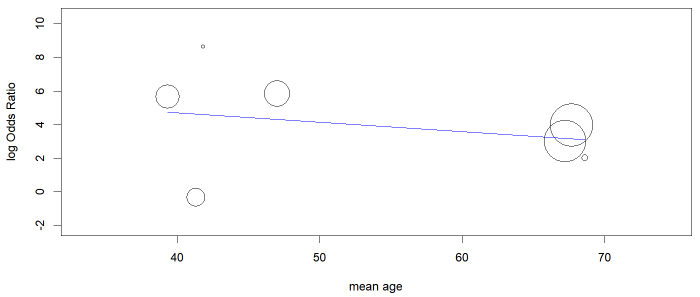


**Supplementary Figure S19**. Meta-regression bubble plot showing the effect of publication year on serious adverse events.


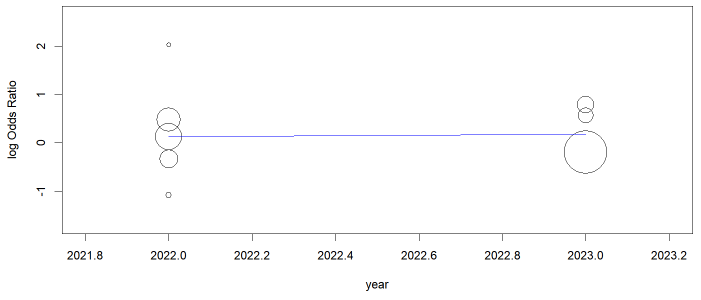


**Supplementary Figure S20**. Funnel plot for serious adverse events.


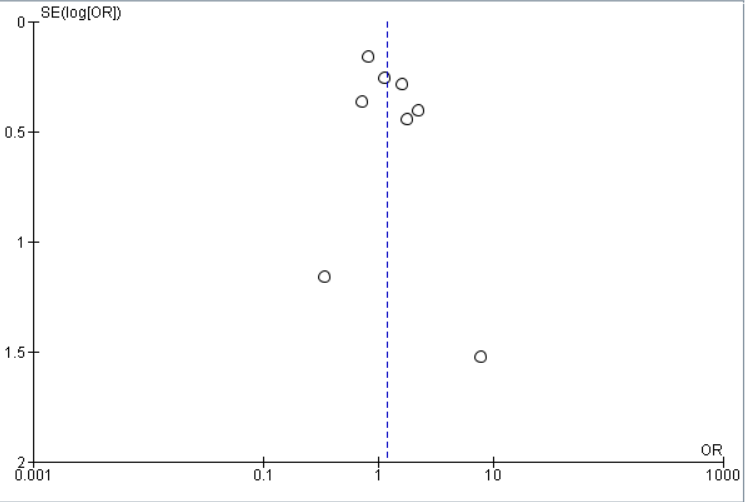


**Supplementary Figure S21**. Subgroup analysis for the incidence of COVID-19 infection.


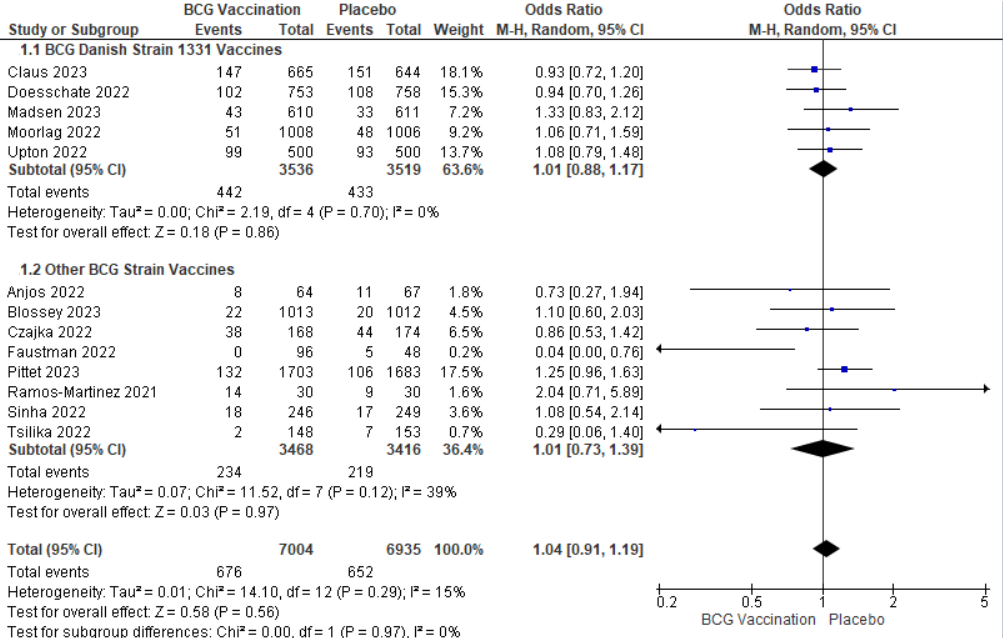


**Supplementary Figure S22**. Subgroup analysis for COVID-19-related hospitalization.


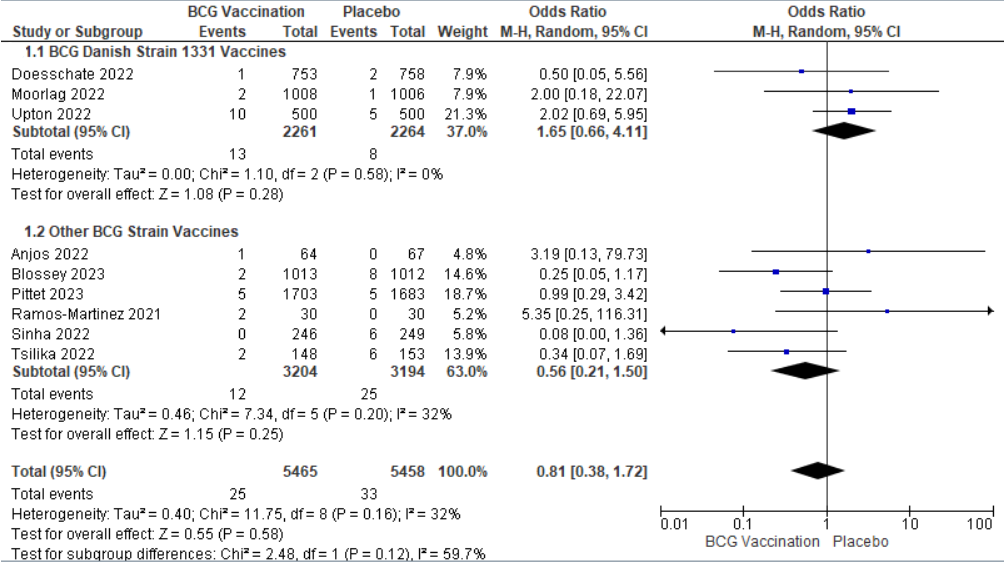


**Supplementary Figure S23**. Subgroup analysis for COVID-19-related ICU admission.


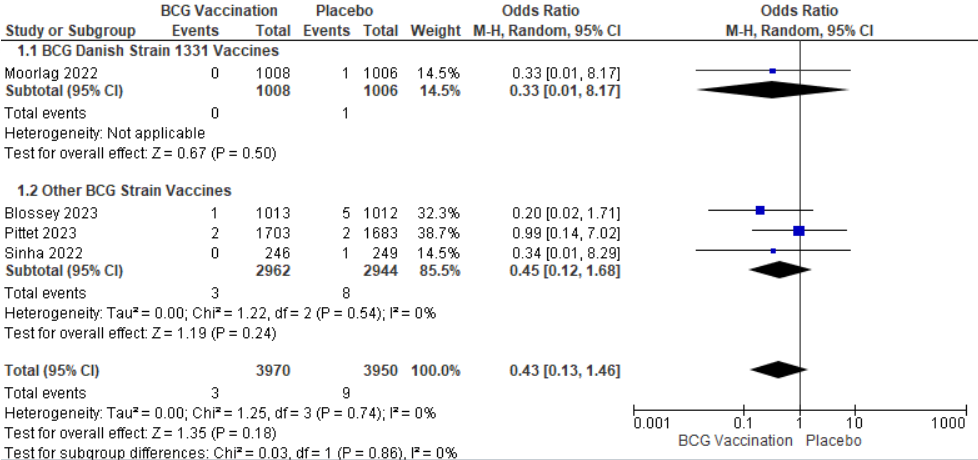


**Supplementary Figure S24**. Subgroup analysis for COVID-19-related mortality.


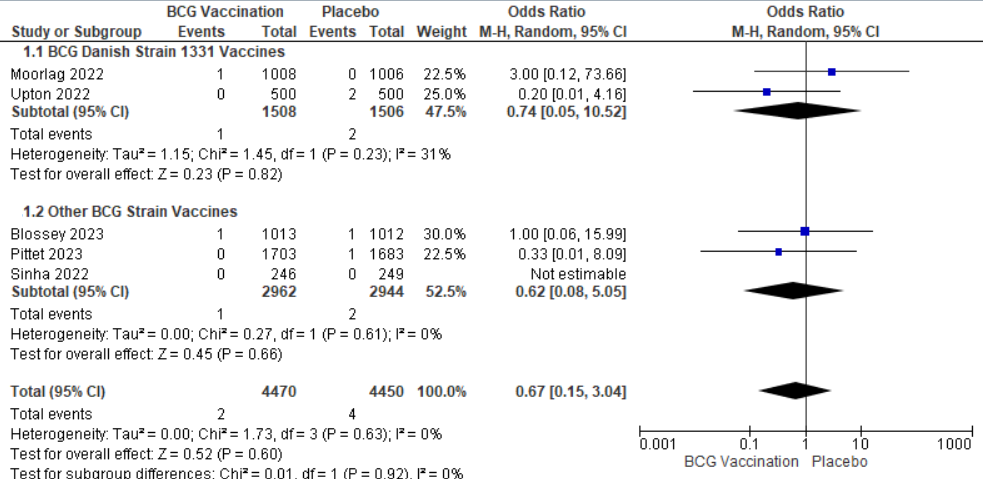


**Supplementary Figure S25**. Subgroup analysis for local injection site complications.


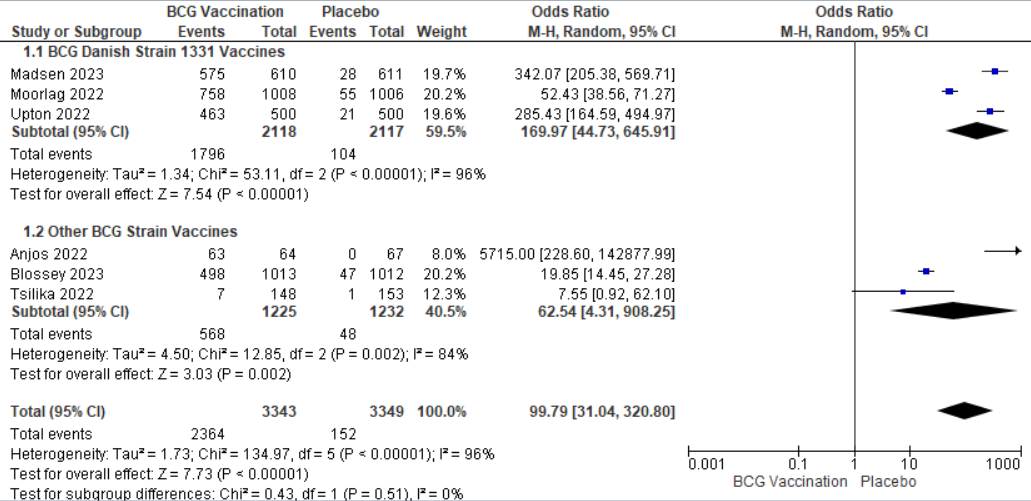


**Supplementary Figure S26**. Subgroup analysis for serious adverse events.


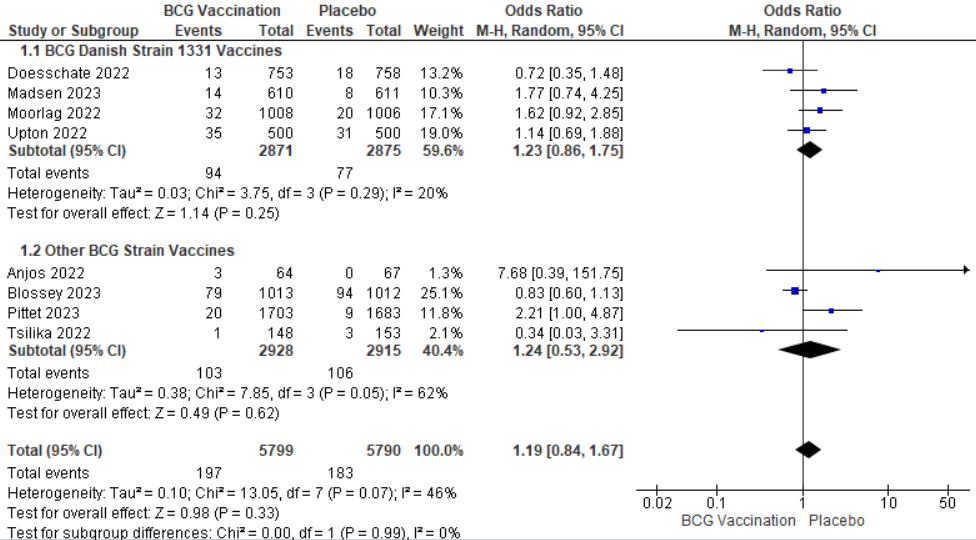


**Supplementary Figure S27**. Cochrane’s Risk of Bias-2 (RoB-2) assessment for risk of bias in randomized controlled trials (RCTs).


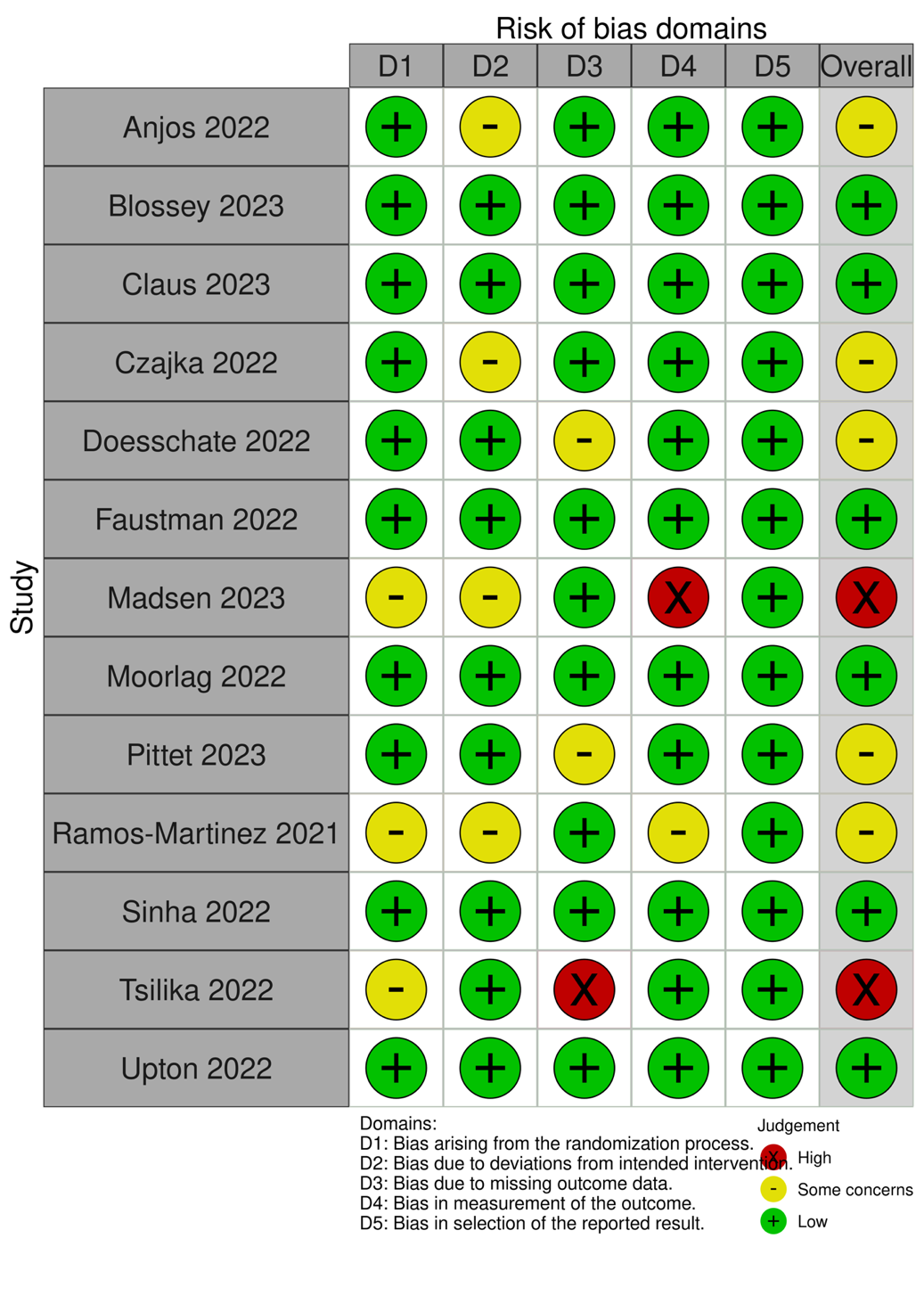


**Supplementary Figure S28**. RoB-2 summary plot.


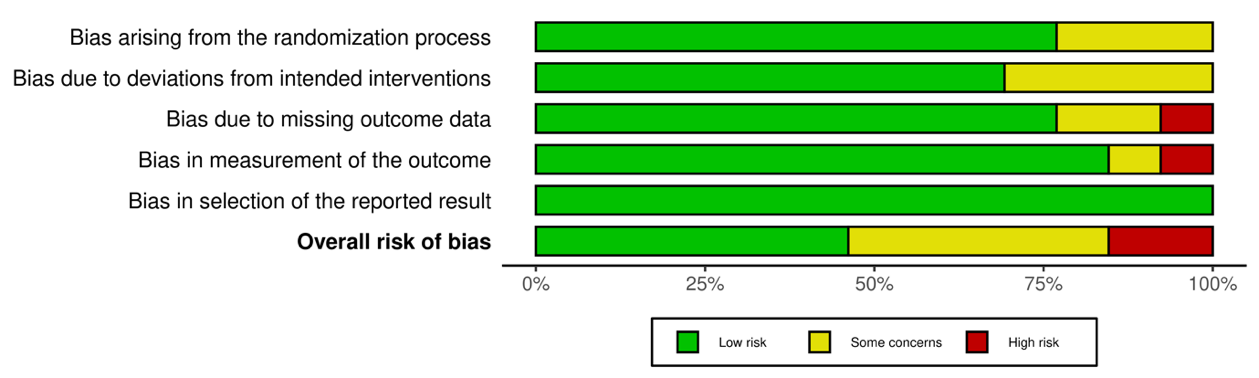

Supplement: Supplementary file 3 [file ms9-86-5439-s003.docx]
